# Influence of hyperthermic intraperitoneal chemotherapy on renal blood perfusion

**DOI:** 10.1007/s00423-023-02948-8

**Published:** 2023-05-24

**Authors:** Lukas F. Liesenfeld, Andreas Brandl

**Affiliations:** Department of General, Visceral and Transplantation Surgery, University Hospital Heidelberg, Im Neuenheimer Feld 420, 69120 Heidelberg, Germany

**Keywords:** HIPEC, Acute kidney injury, Doppler ultrasound, Renal perfusion, Renal resistive index

## Abstract

**Purpose:**

Hyperthermic intraperitoneal chemotherapy (HIPEC) is accompanied with an increased risk of acute kidney injury (AKI). Whether AKI is induced by chemotoxicity or hyperthermia-related changes in renal perfusion remains controversial. The influence of HIPEC on renal perfusion has not been evaluated in patients yet.

**Methods:**

Renal blood perfusion was assessed in ten patients treated with HIPEC by intraoperative renal Doppler pulse-wave ultrasound. Ultrasound (US) examinations were performed pre-, intra-, and postoperative with analyses of time-velocity curves. Patient demographics, surgical details, and data regarding renal function were recorded perioperatively. For evaluation of renal Doppler US to predict AKI, patients were divided in two groups with (AKI +) and without (AKI −) kidney injury.

**Results:**

Throughout HIPEC perfusion, neither significant nor consistent changes in renal perfusion could be observed. Postoperative AKI occurred in 6 of 10 participating patients. Intraoperative renal resistive index (RRI) values > 0.8 were observed in one patient developing stage 3 AKI according KDIGO criteria. At 30 min in perfusion, RRI values were significantly higher in AKI + patients.

**Conclusion:**

AKI is a common and frequent complication after HIPEC, but underlying pathophysiology remains elusive. High intraoperative RRI values may indicate an increased risk of postoperative AKI. Present data challenges the relevance of hyperthermia-derived hypothesis of renal hypoperfusion with prerenal injury during HIPEC. More attention should be drawn towards chemotoxic-induced hypothesis of HIPEC-induced AKI and caution applying regimens containing nephrotoxic agents in patients. Further confirmatory and complementary studies on renal perfusion as well as pharmacokinetic HIPEC studies are required.

**Supplementary Information:**

The online version contains supplementary material available at 10.1007/s00423-023-02948-8.

## Background

Acute kidney injury is a common complication after major abdominal and open thoracic surgeries. After both, the reported risk for occurrence of kidney injury is approximately 15% [1–3]. In specialized surgical centers, selected patients with peritoneal malignancies are treated with cytoreductive surgery (CRS) combined with hyperthermic intraperitoneal chemotherapy (HIPEC) [4]. Dependent on the primary disease, a variety of heterogenous HIPEC regimens and protocols are utilized. In patients undergoing HIPEC treatment, the reported acute kidney injury (AKI) incidence is highly variable with 0.8 to 48% [5–7]. The heterogeneity of reported data and underlying pathophysiology of HIPEC-induced acute kidney injury is not well understood yet. Though multiple factors are likely to contribute to occurrence of kidney injury, two main hypotheses are currently debated: (i) chemo-nephrotoxic related injury and (ii) prerenal injury due to redistribution of blood and relative hypovolemia, derived by hyperthermia and cytokine induced vasodilatation of splanchnic vessels [8, 9]. Several studies reported a significant cohesion between occurrence of AKI and use of cisplatin-containing HIPEC regimen [10–13]. Others were unable to determine any relation of AKI to applied cytostatic drugs or to detect toxic cisplatin plasma levels during perfusion [6, 14–17]. Yet, studies evaluating the influence of HIPEC on renal blood flow in patients are missing. Therefore, aim of the present study was to evaluate if HIPEC has any effects on renal perfusion utilizing renal pulse-wave Doppler ultrasound. Secondary, the capability of renal pulse-wave Doppler ultrasound as a predictor for HIPEC-induced AKI was assessed.

## Material and methods

### Prospective observatory trial

The present study is a single-center prospective observational trial. It was registered at the German Clinical Trial Register (identifier: DRKS00025862; registration date: July 15, 2021) prior first enrollment and was approved by the local ethical committee of the Medical Faculty Heidelberg (S-528/2021). Between July 2021 and December 2022, adult patients with peritoneal malignancies, scheduled for treatment with CRS and HIPEC at the Department of General, Visceral and Transplantation Surgery at the University Hospital Heidelberg, were recruited. Exclusion criteria were (1) patients under the age of 18; (2) HIPEC was canceled or not executed; (3) anatomical or habitual variants hindering sufficient ultrasound performance. Written informed consent was obtained from all patients before enrollment into the study. Data concerning patient demographics, medication, pre-existing illnesses, disease, perioperative, and surgical details were obtained.

**Fig. 1:**
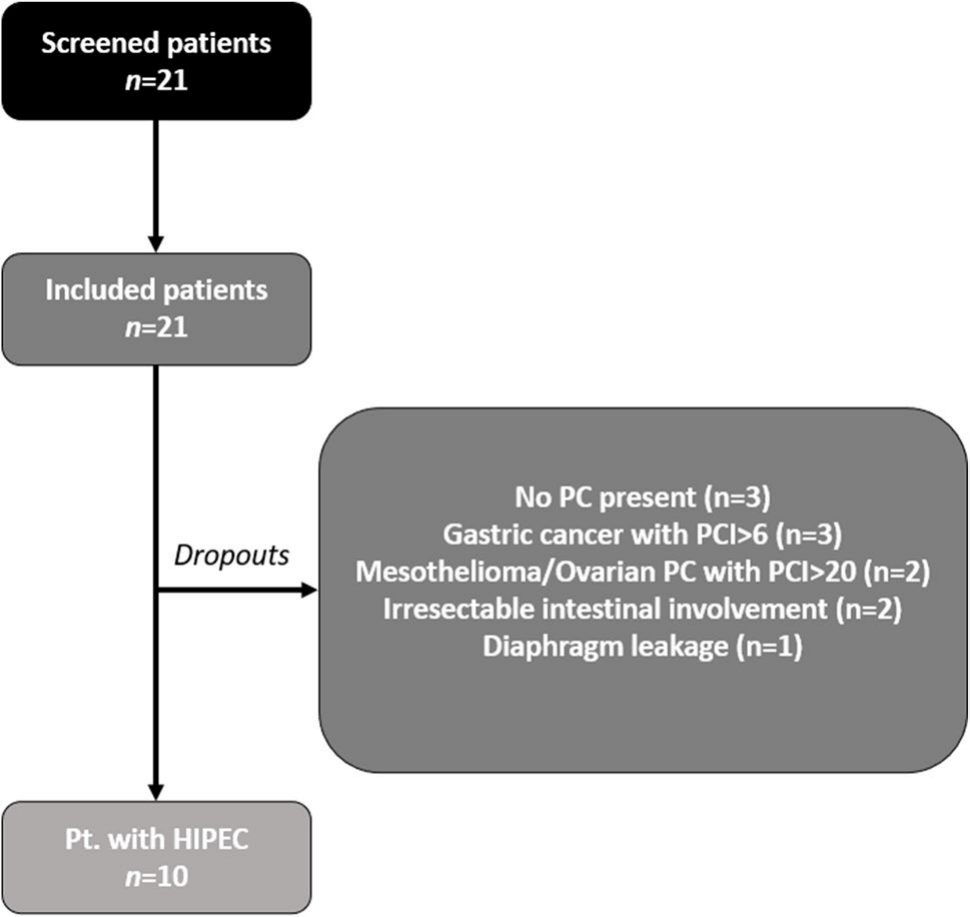
Flow chart diagram of study cohort

Thus, HIPEC procedures were cancelled due to multiple causes, eleven patients (55%) dropped out of this study. PC was not present in three patients, PCI was > 6 in three patients with gastric PC, PCI was > 20 in two patients with ovarian PC, two patients had complete and unresectable involvement of the intestine and in another patient perfusate leaked into thoracic cavity through post-peritonectomy diaphragm sutures (Fig. 1).

### Cytoreductive surgery and hyperthermic intraperitoneal chemotherapy

Indications for CRS and HIPEC were done by multidisciplinary tumor board. All patients underwent exploratory laparotomy and CRS if macroscopic peritoneal tumor nodules were present. HIPEC was applied in closed fashion if complete cytoreduction (completeness of cytoreduction score ≤ 1) could be achieved and peritoneal carcinomatosis index (PCI) did not exceed a value of 20 in patients with ovarian PC or 6 in patients with gastric PC [18–20]. Patients with PMP and mesothelioma were treated with HIPEC whenever complete cytoreduction was achieved. For perfusion, doxorubicin (DOX; 15 mg/m^2^) and mitomycin C (MMC; 15 mg/m^2^) was applied in patients with appendiceal neoplasm, cisplatin (CDDP; 50 mg/m^2^) and MMC (15 mg/m^2^) for ovarian PC, DOX (15 mg/m^2^) and CDDP (50 mg/m^2^) for peritoneal mesothelioma, and oxaliplatin (OXA; 250 mg/m^2^) for gastric PC.

A performer-HT (RanD; Medolla, Italy) peristaltic pump was used for perfusion with an inflow temperature of 42 to 43 °C and a perfusion duration of 90 min. In general, two inflow and outflow catheters have been utilized. The perfusion flow rate was 1 l/min and perfusate volume was 5000 ml. Sodium-chloride (0.9%) was used as solvent and dextrose (1.5%) as perfusate. The extent of parietal peritonectomy was assessed utilizing a parietal peritonectomy score (PPS) by dividing the parietal peritoneum in seven distinct areas, as previously described [21]. Throughout perfusion, head and back of patients were cooled utilizing a cooling cap (HiloTherm; Argenbuehl-Eisenharz, Germany) and cooling mat (Hico-Variotherm 550; Cologne, Germany). Body core temperature (BCT) was tracked using an intra-esophageal placed thermometer.

### Acute kidney injury

To evaluate for the capability of renal pulse-wave Doppler ultrasound to predict HIPEC-induced AKI, the patient cohort was divided in patients with (AKI +) and without (AKI −) AKI. Patients with potential nephrotoxic causes (f. e. obstructive or septic) other than CRS and HIPEC were excluded from this part of analysis. AKI was defined by serum creatinine and urine output criteria (UO) according to the latest Kidney Disease Improving Global Outcomes (KDIGO) guidelines (AKI stage I: increase in serum creatinine > 0.3 mg/dl within 48 h or UO < 0.5 ml/kg/h during 6 h; stage II: increase in creatinine 2–2.9 times baseline or UO < 0.5 ml/kg/h during 12 h; stage III: increase in serum creatinine > 4 mg/dl or 3 times baseline, or UO < 0.3 ml/kg/h during 24 h, or renal replacement therapy) [22]. Serum creatinine was analyzed daily until POD 6 and intra- and postoperative urine output were assessed until POD 2. Fluid balance was tracked on the day of surgery as well as on POD 1 and 2. Intraoperative blood loss and dose of vasoactive drugs during HIPEC perfusion were analyzed. No nephroprotective agents were routinely applied in patients.

### Pulse-wave renal Doppler ultrasound

**Fig. 2:**
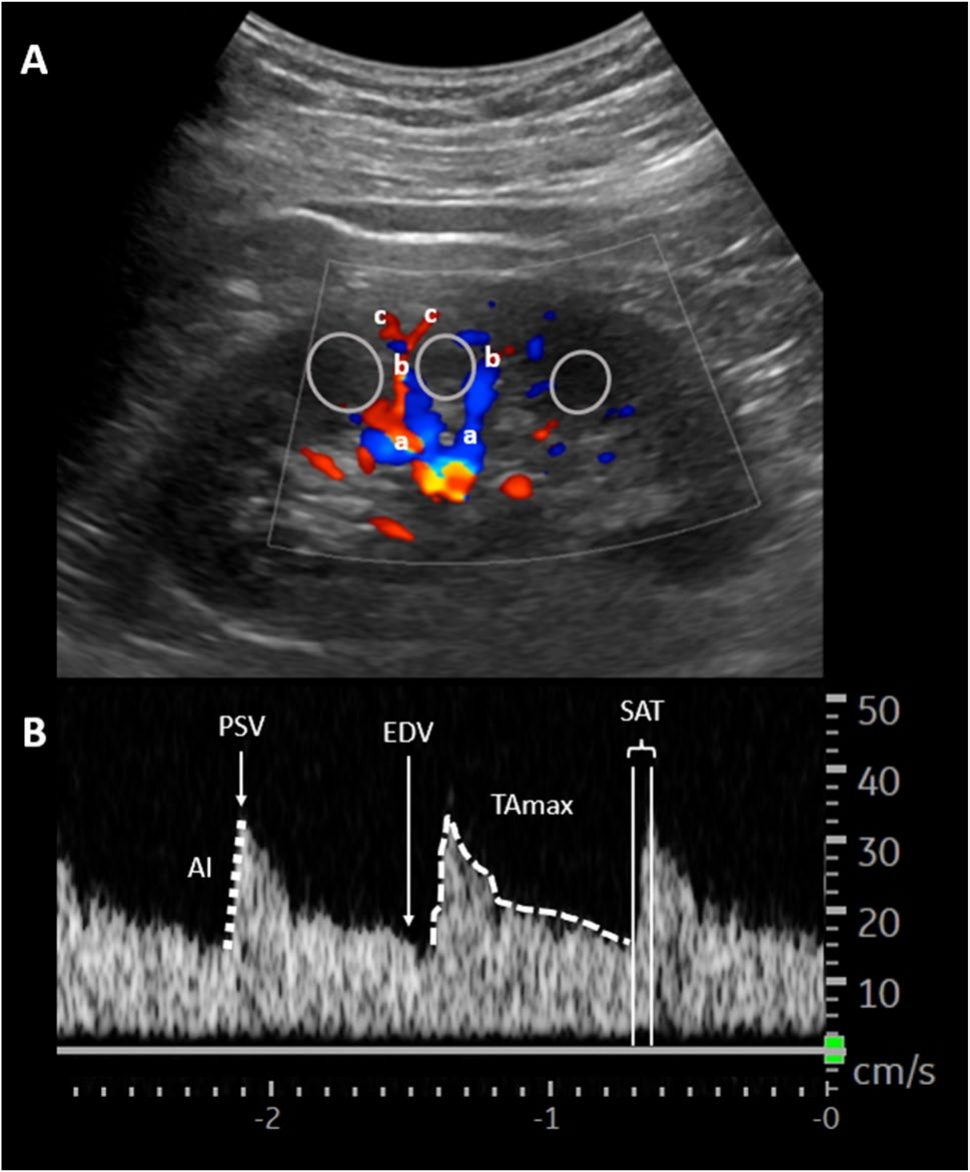
**A** Long axis view of Doppler renal ultrasound and **B** spectral wave form of interlobar artery. Pyramids/medulla (grey circles), segmental vessels (a), interlobar vessels (b) and arcuate vessels (c). Acceleration index (AI), peak systolic velocity (PSV), end-diastolic velocity (EDV), time-averaged maximum velocity (TAmax), and systolic acceleration time (SAT)

Pre-, intra-, and postoperative renal ultrasound was performed using a LOGIQ P9 (General Electric; Boston, MA, USA) ultrasound system with a convex probe (C1-5-RS, GE Healthcare). B-mode was used to measure length and to assess morphology of both kidneys. Renal perfusion was analyzed utilizing pulse-wave Doppler ultrasound and time-velocity curve analyses of interlobar (adjacent to medullary pyramids) or arcuate (at corticomedullary junction) arteries (Fig. 2A, B). Wherever applicable, it was tried to repeat the examinations at the exact same vascular levels in each patient. Preoperative pulse-wave analysis was performed on both kidneys to assess for relevant side differences and to exclude vascular pathologies. Besides, preoperative computer tomography scans were reviewed for relevant renal and vascular pathologies. Twelve intraoperative Doppler ultrasound examinations were performed: prior and at point in time of application of cytostatic drugs, every 10 min after initiation of perfusion as well as after rinsing of abdomen. Last examination was done 1 day after surgery. Ultrasound examinations were performed with patients in supine position. Intraoperative examinations were done under sterile conditions in right or left flank acoustic windows below the sterile drapes.

Time-velocity curves were analyzed for peak systolic velocity (PSV), end-diastolic velocity (EDV), resistive index (RI), systolic acceleration time (SAT), acceleration index (AI), and time-averaged maximum velocity (TAmax). Doppler parameters were measured manually but computed automatically by preinstalled manufacturers’ software. Therefore, interpretation of time-velocity curves was done by two independent physicians with experience in abdominal ultrasound greater 6 years. All examinations were performed by the same physician to reduce inter-operator variability.

Preoperative blood pressure was measured non-invasive and intra- and postoperative blood pressure invasive at each point in time of Doppler ultrasound examination. Furthermore, body core temperatures and perfusate temperatures were recorded.

### Statistical analysis

Data are given as mean ± standard deviation unless otherwise specified. Distribution of data was evaluated with Shapiro–Wilk test for Normality. The nonparametric Mann–Whitney *U* test was used to compare means between the two groups AKI + and AKI − . If variables were dichotomous, Chi-squared test was used. The correlation between two variables was assessed graphically with scatter diagrams and analytically using Pearson correlation coefficient. To evaluate time-dependent effects of HIPEC on renal Doppler parameters, non-parametric Friedman-test have been used. Bonferroni correction was applied when Friedman-test resulted in significancy. Data was computed using BM SPSS software (version 22.0; SPSS Inc., Chicago, IL), and *p* values < 0.05 were considered significant.

## Results

### Patient, disease, and surgical characteristics

**Fig. 3:**
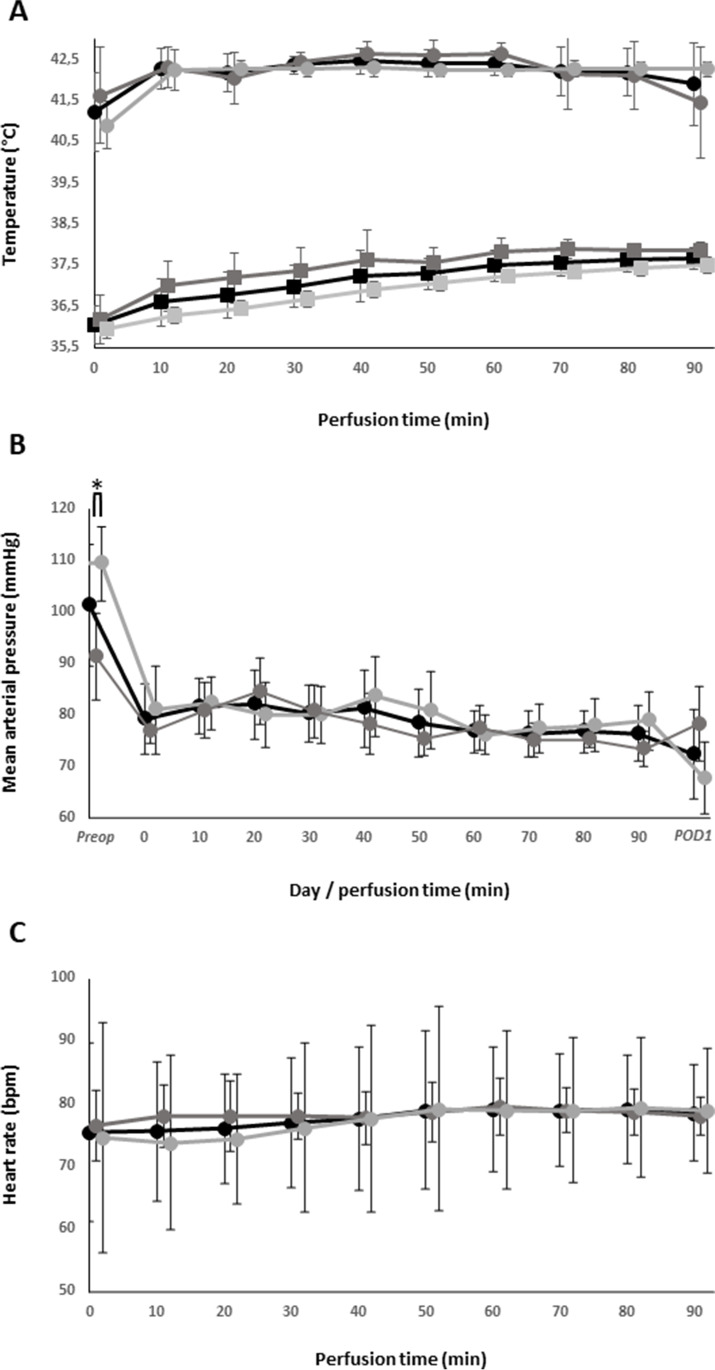
**A** Intraoperative perfusate (circles) and body core (squares) temperatures, **B** pre-, intra- and postoperative mean arterial pressures, and **C** intraoperative heart rates during HIPEC. All patients (*n* = 10) displayed in black, AKI- patients (*n* = 4) in dark grey and AKI + patients (*n* = 6) in light grey. Error bars represent one standard deviation. *p < 0.05

Complete perioperative renal-Doppler ultrasound data was acquired in ten patients treated with CRS and HIPEC (Tables 1 and 2). Four patients underwent HIPEC due to PMP of appendiceal origin and two patients each for mesothelioma and PC of gastric and ovarian origin, respectively. Except one patient with mesothelioma who had recurrent disease and was treated with HIPEC beforehand, all other patients had synchronous peritoneal disease. Both patients with gastric PC completed four cycles of neoadjuvant FLOT-scheme, meanwhile one received nivolumab additionally, and both patients with ovarian PC completed six cycles of neoadjuvant carboplatin and paclitaxel, whereas one patient was additionally treated with avastin. There were more female patients (*n* = 7, 70%) with a mean age of 58 ± 13 years. Body weight was 70.5 ± 16.4 kg, body height 169.5 ± 13.2 cm and BMI 24.6 ± 5.6 kg/m^2^. Chronic kidney disease (CKD) was present in one patient who suffered from AKI induced by priorly applied cisplatin containing HIPEC. Pre-existing congestive heart failure, diabetes mellitus, angiotensin-II-receptor blockers, or relevant renovascular pathologies were not present in any patient. PCI ranged from 0 to 31 (median 13, interquartile range (IQR) 16). Though, PC was not macroscopically present in one patient with gastric cancer; this patient had undergone external laparoscopy with histologically proven PC (PCI = 7) with neoadjuvant therapy and was therefore treated with HIPEC regardless. Completeness of cytoreduction was achieved in all patients; meanwhile, two patients suffering from mesothelioma had a CC-score of 1 due to intestinal involvement. Operation times (laparotomy to final closure) were 312 ± 103 min with median (± IQR) PPS of 2 ± 4. Due to negative pressure warning of the peristaltic pump, the perfusate volume was increased in two patients to 6000 ml and 6500 ml, respectively. Perfusion flow rate was kept constant at 1 l/min in all patients. Targeted perfusion temperatures were reached in all patients at start or within 10 min after application of cytostatic drugs and, with one exception, had been kept constantly above 42 °C (*p* = 0.012; Fig. 3A). In one patient, perfusion temperature dropped just below 42 °C 70 min after start of perfusion. From start of perfusion, BCTs increased slowly but steadily (*p* < 0.001; Fig. 3A). In a single patient at one point in time during perfusion, body core temperature exceeded 38.5 °C.

**Table 1:** Part I — Patient and disease characteristics

Patient	Sex	Age	BMI	Tumor	PC	PCI	Neoadjuvant (cycles)	CKD	Postoperative AKI*	Complications	Surgery time (min)	CC score	PPS
#1	F	76	26,4	Ovary	Syn	20	CBP + PTX (6)	-	1	Delir, wound dehiscence	344	0	7
#2	F	57	21	Appendix	Syn	13	CBP + PTX + B VZ (6)	-	-	-	190	0	3
#3	F	67	20,3	Ovary	Syn	1	-	-	-	Femoral nerve lesion	182	0	1
#4	F	54	24,2	Appendix	Syn	28	-	-	1	UTI	373	0	6
#5	M	35	27,3	Mesothelioma	Recur	12	-	+	3	-	278	1	0
#6	F	65	39,5	Appendix	Syn	8	-	-	1	-	377	0	3
#7	F	63	25,6	Gastric	Syn	0	FLOT + NIVO (4)	-	3	AL, viral encephalitis	296	0	0
#8	F	36	19,3	Gastric	Syn	5	FLOT (4)	-	-	UTI	219	0	1
#9	M	51	21,6	Appendix	Syn	31	-	-	-	Pneumonia	545	0	7
#10	M	72	21	Mesothelioma	-	17	-	-	2	-	315	1	1

*F* female; *M* male; *BMI* body mass index; *PC* peritoneal carcinomatosis; *Syn* synchronous; *Recur* recurrence; *PCI* peritoneal carcinomatosis index; *CBP* carboplatin; *PTX* paclitaxel; *BVZ* bevacizumab; *FLOT* fluorouracil, leucovorin, oxaliplatin, docetaxel; *NIVO* nivolumab; *CKD* chronic kidney disease; *UTI* urinary tract infection, *AL* anastomotic leak; *CC* completeness of cytroreduction; *PPS* parietal peritonectomy score; -: not present; + : present; *staged according to KDIGO classification

**Table 2:** Part II – Intraoperative details and postoperative fluid balance — part II

Patient	IO fluid influx (ml)	IO urine output (ml)	IO blood loss (ml)	IO noradrenaline^#^ (μg)	IO transfusion	IO pRBC (bags)	IO FFP (bags)	POD1 fluid balance (ml)	POD2 fluid balance (ml)
#1	6500	1320	1200	320	Yes	1	4	− 475	188
#2	5850	670	1200	252	Yes	0	2	346	50
#3	5800	820	600	210	Yes	0	2	1680	− 1600
#4	6900	500	2400	381	Yes	2	5	1107	− 264
#5	3600	480	400	1034	No	0	0	− 1076	117
#6	4400	450	600	684	No	0	0	275	− 430
#7	6400	1400	800	505	Yes	0	2	403	− 1706
#8	4800	1000	500	425	No	0	0	241	1845
#9	7000	1940	1000	901	Yes	0	6	− 715	282
#10	4400	570	300	195	Yes	0	4	352	− 1974

*IO* Intraoperative; *pRBC* packed red blood cells; *FFP* fresh frozen plasma; *POD* postoperative day; ^#^received during HIPEC perfusion

Mean arterial pressures (MAP) were significantly higher pre- than intraoperative in all patients. From start to end of perfusion MAPs did not change significantly over time and were constantly above 70 in all patients at any time (*p* = 0.35) (Fig. 3B). Measured heart rates increased slowly and steadily but significantly over time in all patients during perfusion (*p* < 0.001) (Fig. 3C).

Throughout HIPEC, all patients received vasopressor support with 491 ± 278 μg of noradrenaline (range 195–1034 μg). Transfusion of fresh frozen plasma (FFP) was done in seven patients, whereof two additionally got packed red blood cells (pRBC). Total intraoperative fluid influx was 5565 ± 1127 ml (range 3600–7000 ml) with mean urine output of 915 ± 472 ml (range 450-1940 ml) and mean estimated blood loss of 900 ± 583 ml (range 300–2400 ml). On 1st and 2nd POD mean fluid net balances were 214 ± 776 ml (range − 1076 to 1680 ml) and − 349 ± 1093 ml (range − 1974 to 1845 ml), respectively.

Revision surgery was required in one patient with colon anastomotic leakage. Other postoperative complications observed were pneumonia (*n* = 1), delirium (*n* = 1), prolonged laparotomy wound healing (*n* = 1), viral encephalitis (*n* = 1), femoral nerve neuropathy (*n* = 1), and urinary tract infection (*n* = 2).

### Renal Doppler pulse-wave ultrasound

**Fig. 4:**
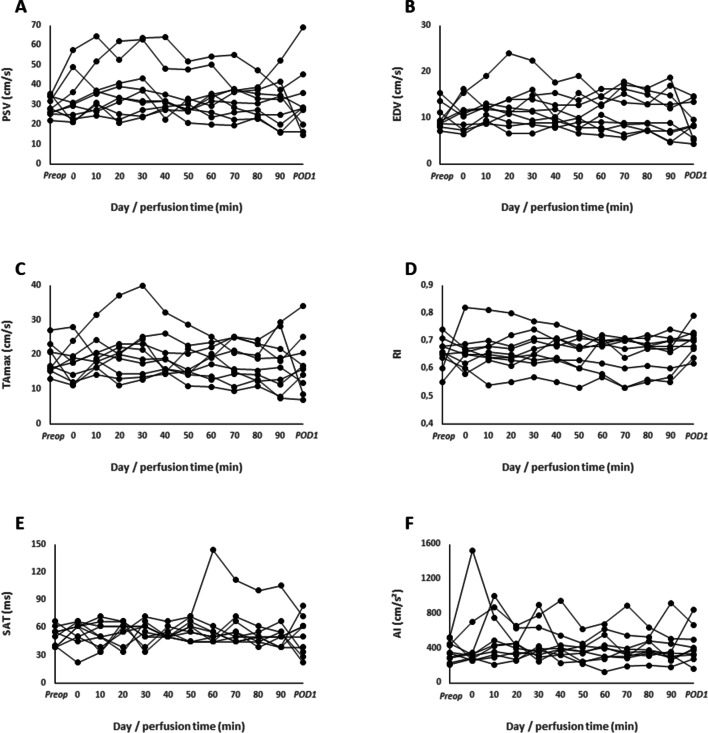
Pre-, intra- and postoperative renal Doppler time-velocity curve analyses. **A** Peak systolic velocity, **B** end-diastolic velocity, **C** resistive index, **D** systolic acceleration time, **E** acceleration index, and **F** time-averaged maximum velocity of *n* = 10 patients (each curve represents one individual)

Two patients presented with a normal variant of an ampullary type of renal pelvis and one patient with a horseshoe kidney. The mean kidney length was 10 ± 0.5 cm. In preoperative examinations, no relevant side differences in renal Doppler waveforms or parameters were present. Thus, right kidneys were chosen to be examined intraoperatively as ultrasound accessibility of right kidneys was appropriate in all patients. Preoperative Doppler waveforms demonstrated normal with a rapid upstroke in systole and a low resistance waveform with continuous forward flow. RIs were just slightly above 0.7 in two patients (0.71 and 0.74). After initiation of perfusion, PSVs increased in two patients approximately by twofold from baseline up to 63 and 64 cm/s, whereof one patient had increased EDV of 24 cm/s (approx. 2.6-fold from baseline) as well with consecutive increase in TAmax half through perfusion duration (*p* > 0.05) (Fig. 4A, B, C).

High-resistance waveform patterns occurred in one patient. The RI was 0.82 at start of HIPEC but steadily returned to normal values throughout and till the end perfusion (Fig. 4D). On POD1, one patient presented with an increased RI to 0.79. Increased RI’s neither correlated with increased or decreased heart rates, MAP’s, perfusion temperatures, body core temperatures nor systolic or diastolic blood pressures at any time. SAT values showed modest variations throughout perfusion, but in a single patient, a sudden increase at 60 min after perfusion was measured (Fig. 4E). Through first half of perfusion mean SAT was 64 ms in this patient but increased to a maximum of 144 ms followed by slow decline. Values did not return to normal at end of perfusion (105 ms) but on POD1 (72 ms).

Low baseline preoperative AI values (< 300 cm/s^2^) were measured in two patients which did not vary remarkably intraoperatively. In three patients, distinct increase from baseline and high variability in AI values were observed (Fig. 4F). Neither PSV, EDV, TAmax, RI, SAT, nor AI values changed significantly through time of perfusion (*p* > 0.05).

### Postoperative acute kidney injury

According to KDGIO, AKI occurred in six patients (60%) whereof three patients suffered stage 1, one patient stage 2 and two patients’ stage 3 renal injury. Renal replacement therapy was not required in any patient. Septic disease due to anastomotic leakage could not be excluded as cause of AKI in one patient with stage 3 renal injury and was therefore excluded from this part of analysis. Exclusion of one patient resulted in five patients being in AKI + and four patients in AKI- group. In all five patients in which AKI occurred, positive KDIGO criteria (increase in serum creatinine levels) were met on 2nd POD (Suppl. Fig. 1).

Preoperative MAP values were significantly higher in AKI + patients with 109 ± 7 mmHg vs. 91 ± 8 mmHg in AKI- patients (*p* = 0.032; Fig. 3B). Two out of five patients in AKI + group but none in AKI- group suffered from arterial hypertension. Neither perfusate volume, body core temperature, perfusion temperature, heart rate, amount of noradrenaline during HIPEC, intraoperative transfusion, nor intraoperative fluid balance inclusive fluid balance on 1st and 2nd POD were significantly different between patients with and without AKI.

**Fig. 5:**
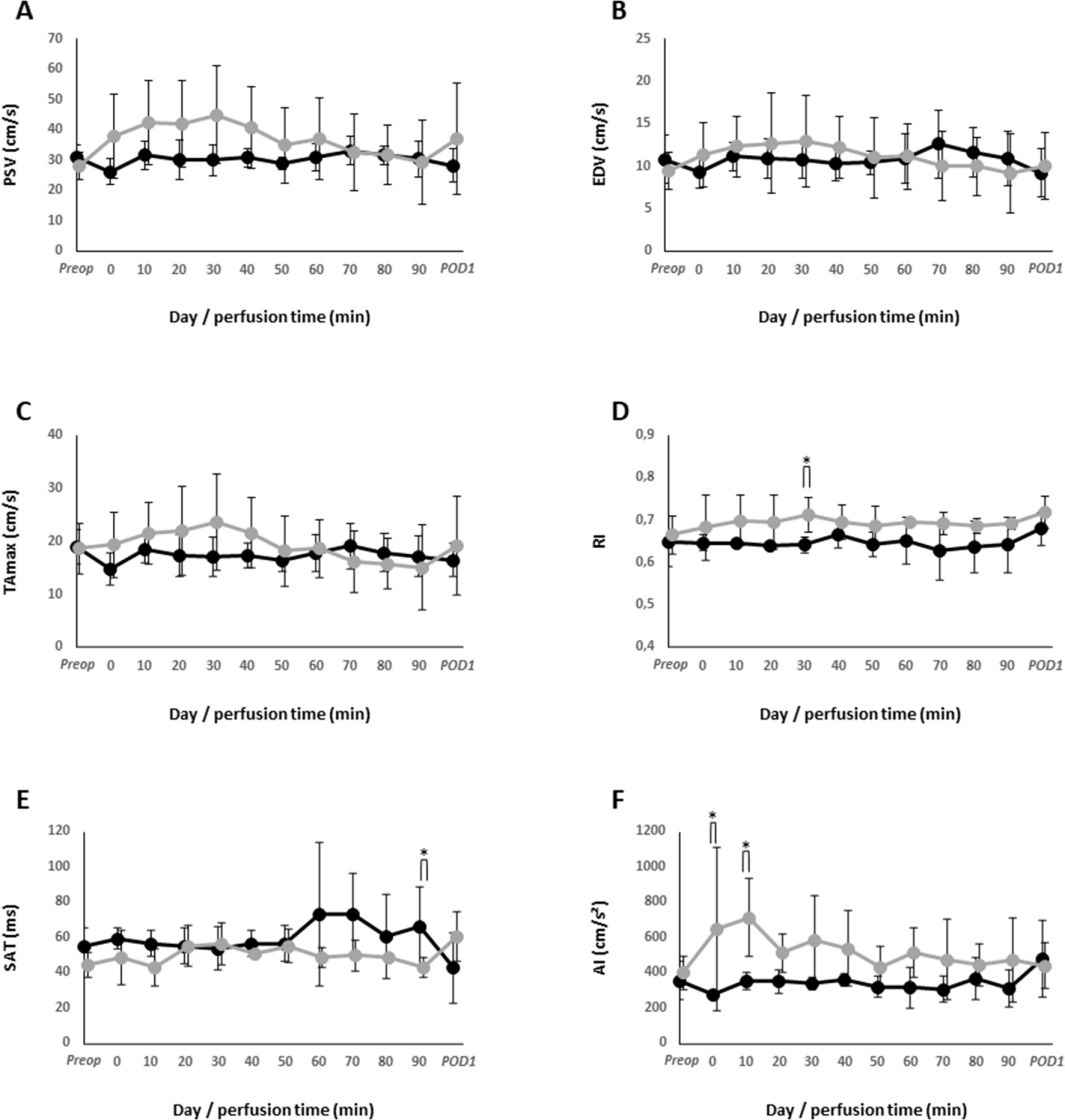
Intraoperative renal Doppler parameters during HIPEC in patients (*n* = 9) with (grey) and without (black) AKI. **A** Peak systolic velocity, **B** end-diastolic velocity, **C** resistive index, **D** systolic acceleration time, **E** acceleration index, and **F** time-averaged maximum velocity. Circles represent mean values and error bars represent one standard deviation. *p < 0.05

Renal pulse-wave Doppler parameters of AKI + and AKI- patients are shown in Fig. 5. On average, RI values were higher in AKI + patients independent of points in time but reached statistical significance exclusive at 30 min within perfusion with 0.71 ± 0.04 in AKI + and 0.64 ± 0.02 in AKI- patients (*p* = 0.032; Fig. 5D). SAT was significantly lower in AKI + patients at end of perfusion (90 min) with 43 ± 10 vs. 67 ± 22 ms in AKI- patients (*p* = 0.032; Fig. 5E). At start of perfusion and 10 min after initiation, AI values were significantly higher in AKI + patients (651 ± 463 vs. 278 ± 23 cm/s^2^ and 715 ± 220 vs. 356 ± 47 cm/s^2^, respectively; at each timepoints *p* = 0.016; Fig. 5F). Independent of points in time, neither PSV, EDV nor TAmax was different between both groups.

## Discussion

Combinative treatment of CRS and HIPEC is yet the established treatment for selected patients with peritoneal malignancies (PM) as it offers best outcomes. Nonetheless, CRS and HIPEC are accompanied by relevant morbidity due to typically major inevitable surgical trauma and concomitant chemotoxicity [23, 24]. Common postoperative complication is acute kidney injury with incidence up to 48% [6]. Pathophysiological mechanisms causing HIPEC-induced AKI are not well defined yet.

Systemic exposure to nephrotoxic drugs such as cisplatin, which is frequently utilized for HIPEC in patients with ovarian and gastric PC as well as with mesothelioma, could be one explanatory cause [12, 25]. Amongst other studies, we identified cisplatin-containing perfusion regimens as the strongest independent risk factor for occurrence of HIPEC-induced AKI [13]. Contradictory, other studies were unable to identify any relationship between use of cisplatin and kidney injury [10, 15, 16]. In the, to date, most suitable multicentric randomized-controlled clinical trial to prove cisplatin-containing HIPEC to be beneficial for selected patients with ovarian PM, none of the participating patients developed postoperative AKI [26]. It has to be respected, all patients received sodium-thiosulfate in nephroprotective intent and utilized cisplatin concentrations in perfusate were not reported. Thus, supported by pharmacokinetic studies in which cisplatin plasma levels in patients were below the cytotoxic threshold, led to alternative hypothesis of cytokine- and hyperthermia-derived AKI [8, 17]. Mimicking a septic-like state, hyperthermia, and surgical trauma–related cytokines are thought to induce musculocutaneous and splanchnic vasodilatation with severe blood volume redistribution issues resulting in renal hypoperfusion (prerenal kidney injury) [8, 9]. To which extent these mechanisms are actual relevant and involved in the pathophysiology of HIPEC-induced AKI, remain elusive. We were unable to observe any renal injuries derived by hyperthermic intraperitoneal perfusion in a preclinical rodent HIPEC model [13]. Therefore, we conducted the present study to evaluate the influence of HIPEC on renal perfusion in humans. To our best knowledge, we are first to evaluate and report on the influence of HIPEC on renal perfusion in patients.

Distinct trends from preoperative to intraoperative Doppler values as well as variations throughout perfusion could be observed but were exclusive and inconsistent to one to two patients dependent on the studied Doppler parameter and were without statistical significancy over time. Preoperative MAP values were significantly higher but not pathological hypertensive in AKI + patients. In our most recent retrospective analysis, patients with arterial hypertension were not exposed to have increased risk for occurrence of HIPEC-induced AKI [13]. Kidneys adapted to relative higher blood pressures are potentially more vulnerable to low blood pressures but it is more likely to be an incidental finding caused by the cohort size.

High resistances with RI > 0.8 were present in one patient, but already at initiation of perfusion. Thus, this alteration in renal perfusion cannot be contributed to effects of hyperthermia. In the middle of perfusion, one patients SAT showed a sudden twofold increase which could be secondary to hyperthermia but were without concomitant changes in BCT, MAP, and HR. Renal resistive index (RRI) and systolic acceleration time (SAT) are commonly utilized Doppler parameters to evaluate for renovascular pathologies and renal blood perfusion [27–30]. Unlike PSV, EDV, Acc, and TAmax, both, RI and SAT, are independent of Doppler angle and are therefore less intra- and interobserver variable [31, 32]. RI values > 0.7, SAT values > 70 ms and Acc values < 300 cm/s^2^ are considered abnormal, but these cutoffs were defined in patients at states which are very different to those under operative circumstances [27, 33]. Mean pre-, intra-, and postoperative RRI values were higher in AKI + patients, but statistical significancy was reached solely at 30 min after perfusion. Our results are unable to prove but may indicate intraoperative RRI to be a potential predictor of postoperative AKI in patients undergoing abdominal surgery with HIPEC. Recently, RRI was increasingly recognized as a potential predictor for postoperative AKI in patients undergoing cardiac surgery measured by transesophageal ultrasound. In these studies, intraoperative defined RI cutoff values for positive likelihood of AKI to occur, were greater 0.74 or 0.75, respectively [34, 35]. Thus, usually applied normal values cannot be transferred to circumstances within the operating room. It has to be considered, RI is not truly reflecting renal blood perfusion but is not less than a complex product dependent on interactions of several factors such as renal interstitial pressure, vascular resistance, and compliance as well as systemic hemodynamics [36–38]. Therefore, validity of one universal RI cutoff value for all renal pathologies is doubted by professionals [39]. At one and two timepoints, both, SAT and AI, were significantly different between AKI + and AKI- patients. AI values were higher and SAT values were lower in AKI + patients which, in fact, both, from renal Doppler US point-of-view, represent more physiological values. Thus, we rather tend to interpret these changes unspecific than to contribute them to harmful hyperthermia-derived effects.

In patients undergoing abdominal surgery, data on intraoperative renal Doppler parameters have not been published yet. This is most likely due to practical issues. Surgery would have to be interrupted to obtain images of the kidneys and the usual scanning region lies often within the sterile field. Reports on serial intraoperative renal Doppler measurements, equivalent to our current study, are missing. Therefore, interpretation of our data is limited by lack of experience and availability of comparative data. In accordance with studies on patients undergoing cardiac surgery, the patient with highest RI in our cohort developed the most severe postoperative AKI (stage 3 according to KDIGO criteria) [35]. The applicability of the present data is limited by overall small sample size and small number of patients with severe HIPEC-induced AKI (one patient with stages 2 and 3 AKI each). Especially regarding our secondary aim, the capability of renal Doppler ultrasound to predict AKI; these limitations prohibit relevant conclusions. Thus, our results on predictability are more of indicative character.

Contrast-enhanced renal ultrasound (CEUS) is an advantageous and complementary technique to evaluate renal blood perfusion in patients but would have been unsuitable in obtaining serial measurements [40]. Though, contrast agents applied in ultrasound are known for their relative safety, cumulative dose required to perform equivalent frequent measurements as in this study, would have been extraordinarily higher than doses used in clinics with unknown side-effects.

Points in time, in which hyperthermia-induced changes of renal blood perfusion as well as compensatory physiological reactions occur, are unknown. Therefore, respecting methodological limitations, renal Doppler ultrasound was method of choice to perform the current study as it allows very frequent and reproducible measurements.

We were unable to identify neither significant nor consistent changes in renal blood perfusion throughout HIPEC perfusion in patients. Thus, our data challenges the hypothesis of hyperthermia-related renal hypoperfusion and whose clinical relevance. The ambiguous literature on chemotoxicity-derived hypothesis of AKI could be explained by rarely reported and highly variable utilized drug concentrations in perfusate as well as other not yet acknowledged pharmacokinetic factors, such as the extent of parietal peritonectomy [21]. However, confirmative complementary prospective clinical studies evaluating renal blood perfusion in HIPEC patients, and further pharmacokinetic HIPEC studies are required, to prove the chemotoxicity-derived AKI hypothesis. At first, knowledge of the underlying pathophysiology of HIPEC-induced AKI is essential to develop effective preemptive strategies and treatments.

## Conclusions

Postoperative AKI is a common complication following CRS with HIPEC, but underlying causes are not yet distinctly defined. We could not identify significant or consistent changes in renal blood perfusion throughout HIPEC perfusion utilizing renal Doppler US in patients. Intraoperative RRI values were higher in patients with AKI and may have potential predictive capability. Thus, hypothesis of prerenal kidney injury derived by hyperthermia-mediated renal hypoperfusion is presumably of minor relevance in pathophysiology of HIPEC-induced AKI. More attention should be paid to HIPEC regimens containing nephrotoxic agents. Confirmatory studies and advanced pharmacokinetic studies are required to unveil factors explaining the currently ambiguous literature on this subject.

### Supplementary Information

Below is the link to the electronic supplementary material.

Supplemental Fig. 1 Preoperative and postoperative serum creatinine values until 6th POD in patients (n=9) with (grey) and without (black) postoperative AKI according to KDIGO criteria. (PNG 198 kb)

High resolution image (TIF 24 kb)

## Data Availability

The data that support the findings of this study are available from the corresponding author, L.F.L., upon reasonable request.
