# Supplementary figures and images for: Influence of hyperthermic intraperitoneal chemotherapy on renal blood perfusion
Source: Langenbecks Arch Surg. 2023 May 24;408(1):207. doi: 10.1007/s00423-023-02948-8 (PMC10209305; doi:10.1007/s00423-023-02948-8)

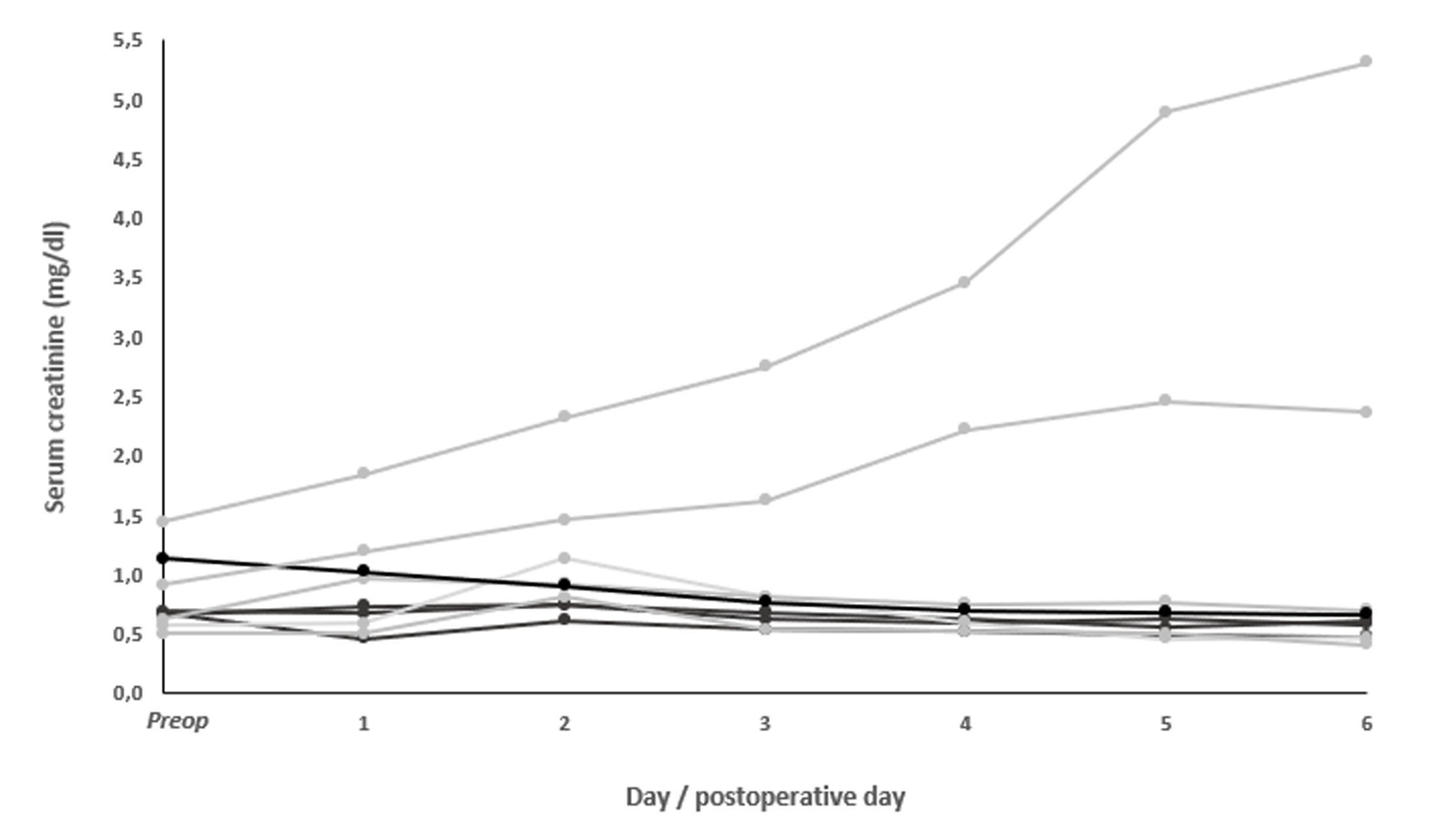

Supplement: Supplementary file 1 — Preoperative and postoperative serum creatinine values until 6th POD in patients (n=9) with (grey) and without (black) postoperative AKI according to KDIGO criteria. (PNG 198 kb) [file 423_2023_2948_Fig6_ESM.png]

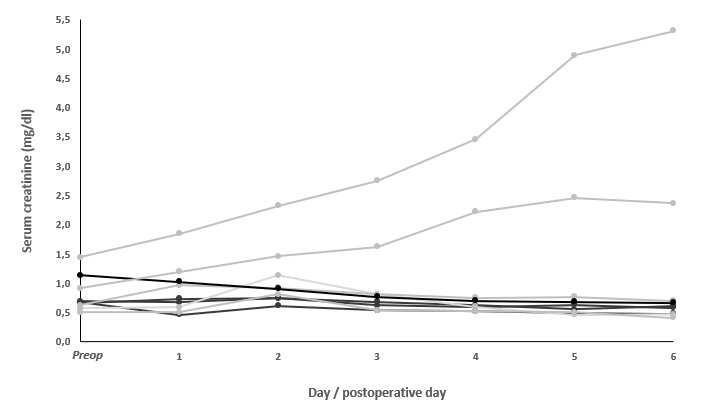

Supplement: Supplementary file 2 — High resolution image (TIF 24 kb) [file 423_2023_2948_MOESM1_ESM.tif]
